# Dubya Echoes Confirmation of Human-Induced Global Warming Theory and an Antioxidant Unravels

**DOI:** 10.1100/tsw.2001.63

**Published:** 2001-06-15

**Authors:** Shauna Haley

## Abstract

U.S. President George W. Bush's nod to scientific evidence for human industrialization's major role in the onset of global climate change grabs both top story positions in Nature and Science this week.

A month ago, a committee of leading U.S. scientists formed by the National Research Council (NRC) began a review of the findings of the Intergovernmental Panel on Climate Change's (IPCC) three-year study on the genesis and perpetuation of global warming. Late last week, the team reported that the conclusions of the IPCC 'accurately reflect current scientific thinking', suggesting strongly that humans are creating a worldwide climate shift, report both *Nature* and *Science*. The short review was requested by the U.S. president after his refusal to implement the international Kyoto Protocol in March. In the face of scientific confirmation of the IPCC report, however, president Bush has called for a new round of research into solutions for slowing the greenhouse effect, saying that science and technology must resolve this problem, reports *Science*. Dubya's stance may prove very unpopular with European governments, though, says *Nature*, noting that the president plans to inform European leaders while there this week that he will not enforce mandatory restrictions on carbon emissions in the U.S., despite the NRC's relative endorsement of the IPCC report. Although the NRC did indicate that the IPCC report could have focused more on unanswered questions about global warming, the general conclusion that humans are probably directly responsible for climate change could further galvanize international sentiment against the U.S. president's treatments of environmental issues. *Nature* cites the statistic that while the U.S. contains only 5 percent of the world's population, it produces 25 percent of all carbon emissions. President Bush, in a perhaps vintage stall-and-appease tactic, has called for a five-year research program, not unlike the decade-long one created by George Sr. that is slated for a budget cut in 2002.

## *Editor's Note:* The Unraveling Antioxidant?

Although TWINS normally only comments on news items, a new scientific report in *Science* has the promise of news-in-the-making. Researchers Lee, Oe and Blair, while examining potential explanations for vitamin C's failure as a prevention measure for cancer, found that the antioxidant may actually contribute to DNA damage. Previous research has demonstrated that certain heavy metals (occasionally found in human blood) in combination with the vitamin create mutagenic compounds. However, evidence for similar genotoxins have also been found when the metals are absent, puzzling physicians. Lee, Oe and Blair, following experiments with non-metal-containing plasmas, found that vitamin C alone may mediate the formation of specific genotoxins that are precursors of highly mutagenic DNA lesions. The new results, while not conclusive, may provide intriguing new insight on the possible molecular activities of antioxidants – and could certainly cause a ruckus in the dietary supplement industry.
